# Fusion of Clinical and Deep Learning Features for Predicting Pembrolizumab Monotherapy Response in Advanced Non-Small Cell Lung Cancer

**DOI:** 10.3390/jcm15124536

**Published:** 2026-06-11

**Authors:** Liton Devnath, Ian Janzen, Cheryl Ho, Barbara Melosky, Stephen Lam, Calum MacAulay, Ren Yuan

**Affiliations:** 1 Basic and Translational Research, BC Cancer Research Institute, 675 West 10th Avenue, Vancouver, BC V5Z Il3, Canada; ijanzen@bccrc.ca (I.J.); slam2@bccancer.bc.ca (S.L.); 2 Department of Pathology, Faculty of Medicine, University of British Columbia, 2329 West Mall, Vancouver, BC V6T IZ4, Canada; 3 Interdisciplinary Oncology Program, Faculty of Medicine, University of British Columbia, 2329 West Mall, Vancouver, BC V6T IZ4, Canada; 4 BC Cancer, Vancouver Center, 600 West 10th Avenue, Vancouver, BC V5Z 4E6, Canada; cho@bccancer.bc.ca (C.H.); bmelosky@bccancer.bc.ca (B.M.); ren.yuan@bccancer.bc.ca (R.Y.); 5 Department of Medical Oncology, Faculty of Medicine, University of British Columbia, 2329 West Mall, Vancouver, BC V6T IZ4, Canada; 6 Department of Respirology, Faculty of Medicine, University of British Columbia, 2329 West Mall, Vancouver, BC V6T IZ4, Canada; 7 Department of Radiology, Faculty of Medicine, University of British Columbia, 2329 West Mall, Vancouver, BC V6T IZ4, Canada

**Keywords:** NSCLC, pembrolizumab, deep learning, PD-L1, immunotherapy, treatment response

## Abstract

**Objective**: Pembrolizumab monotherapy is an anti-PD-1 immunotherapy that is approved as a first-line treatment for non-small cell lung cancer (NSCLC) patients with high PD-L1 expression (≥50%). However, approximately 55% of these patients do not respond. Early identification of likely non-responders is critical to enable timely transition to alternative treatments. **Materials**: This study analyzed a retrospective cohort of NSCLC patients treated with first-line PD-L1 monotherapy, divided into a discovery training set (*n*: 97; 27 non-responders) and a preliminary test set (*n*: 17; 9 non-responders). Treatment response was assessed using baseline and follow-up CT scans in accordance with the response evaluation criteria in solid tumors (RECIST v1.1). **Methods**: Our objective was to extract deep learning (DL) features from the two groups of patients and apply transfer learning techniques to identify patients at risk of progression on pembrolizumab monotherapy. A nonparametric statistical test (Mann–Whitney U) was employed to rank the discriminative power of the 128 features from these training groups. Two types of support vector machine (SVM-RBF and SVM-Polynomial) classifiers were employed to investigate the discriminating power of the highest-ranked features as measured by F1 score and AUC values over ROC curves at the three levels of the data (slice, lesion, and patient) with and without clinical descriptors. **Results**: SVM-RBF performed best when trained on the 10 highest-ranked DL features and five clinical descriptors, achieving AUC of 0.742 (CI 95% 0.47–1.00), SN of 88.9%, SP of 75% and F1 score of 84.2% on preliminary test set patients, whereas an AUC of 0.902 ± 0.031, SN of 81.5%, SP of 81.4% and F1 score of 71% were observed for the discovery training set. **Conclusions**: Integrating CT-based DL features with clinical descriptors demonstrated balanced performance, offering a promising tool to identify patients at risk of progression on pembrolizumab monotherapy to support first-line treatment decisions in PD-L1-high NSCLC.

## 1. Introduction

Lung cancer is the leading cause of cancer-related deaths worldwide, with a 5-year survival rate of approximately 22%, predominantly due to late-stage diagnoses where curative options are limited [1,2]. Non-small cell lung cancer (NSCLC) accounts for the majority of lung cancer cases, and therapeutic strategies for advanced NSCLC are increasingly guided by the identification of actionable genetic alterations, including EGFR, ALK, ROS1, BRAF, KRAS, and NTRK mutations [3,4]. These genomic biomarkers enable precision medicine approaches, offering targeted therapies that have redefined treatment paradigms. For patients without detectable driver mutations, immune checkpoint inhibitors (ICIs) have revolutionized management, consistently outperforming platinum-based chemotherapy in clinical trials, both as monotherapy (e.g., pembrolizumab) and in combination with chemotherapy [5,6,7,8]. Pembrolizumab is endorsed by Health Canada for advanced NSCLC patients exhibiting high PD-L1 expression (≥50%) without EGFR or ALK mutations, a milestone in personalized cancer care. Nevertheless, despite this breakthrough, nearly 55% of this PD-L1-high cohort exhibit stable or progressive disease following pembrolizumab monotherapy, underscoring the critical need for robust predictive biomarkers to distinguish non-responders before treatment initiation [4]. Such stratification would refine first-line therapeutic decision-making, ensuring timely and personalized interventions for improved clinical outcomes. Unfortunately, the absence of well-defined predictive biomarkers or clinicopathological characteristics to identify non-responders to first-line pembrolizumab complicates treatment selection, leaving clinicians without reliable tools to guide therapy decisions [9]. To improve patient outcomes, it is essential to refine first-line treatment strategies and enable timely, personalized interventions.

Within quantitative medical imaging, radiomics and AI-based methodologies apply algorithmic analysis to transform image data into structured numerical representations, facilitating the detection of subtle imaging characteristics that escape standard visual evaluation [10,11,12]. Deep learning (DL)-derived risk scores have shown partial overlap with radiomic features, yet radiomics alone consistently underperformed, indicating that deep learning captures additional imaging patterns beyond standard radiomic descriptors in a study predicting immunotherapy benefit for NSCLC patients [13]. Prior DL-based immunotherapy studies in NSCLC have shown that DL features can be effectively combined with demographic, metastasis, genomic, and clinical variables to build predictive models for diagnosis, prognosis, biological endpoints, and treatment response [14,15,16,17,18,19,20,21,22]. Several studies have also demonstrated that using a selected subset of DL features—rather than the full feature set—can enhance model robustness and promote machine learning classifier diversity [18,20,22]. Notably, two investigations extracted DL features from both PET and CT imaging [19,20], achieving superior performance compared with radiomic and clinical models across training and validation cohorts. However, these studies did not describe how patient-level risk scores were derived from multiple lesions or slice-level predictions, leaving an important methodological gap.

Prior work has examined the predictive value of baseline CT and clinical characteristics, reporting that baseline burden quantified from CT (e.g., number of involved organs) together with smoking exposure (pack-years) are independent predictors of progressive disease in patients treated with pembrolizumab, and may help identify non-responders [23]. Our prior study found that baseline radiomics features derived from conventional CT imaging, together with clinical characteristics, showed a significant association with response to pembrolizumab monotherapy. However, the model recorded a false positive rate of 37% in the preliminary test cohorts, indicating substantial room for improvement [24].

Motivated by prior studies [13,14,15,16,17,18,19,20,21,22], this work integrates deep learning (DL) features with baseline CT-derived clinical variables to develop and validate a digital AI system for improved prediction of PD in patients treated with pembrolizumab monotherapy. In addition, a detailed multi-level analysis—at the slice, lesion, and patient levels—was performed to understand PD risk stratification across different spatial scales.

## 2. Materials and Methods

### 2.1. Dataset Review and Response Assessment

**Figure 1:**
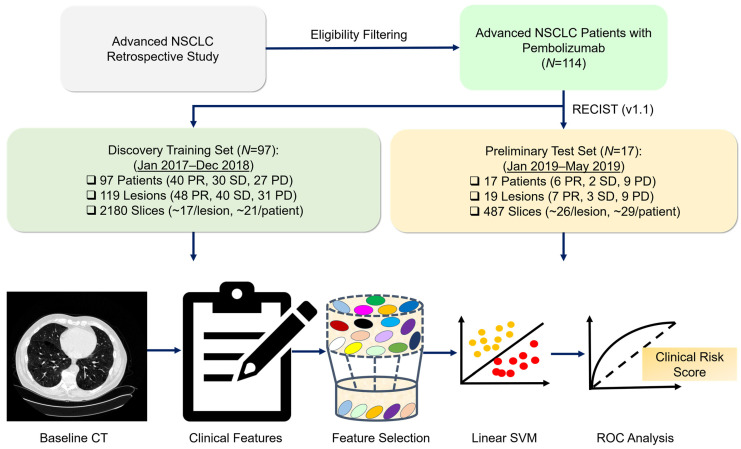
Data processing pipeline and clinical score calculation. A total of 114 patients were selected according to RECIST criteria and subsequently divided into two retrospective cohorts: a discovery (training) cohort and a preliminary test cohort. A clinical risk score was computed using the selected baseline features as inputs to a linear support vector machine classifier.

We retrospectively analyzed a cohort of patients treated at BC Cancer with stage IIIB-IV non-small cell lung cancer lacking EGFR/ALK mutations who received first-line pembrolizumab monotherapy and had PD-L1 expression ≥50% (assessed by an institution standard IHC assay). Eligible patients were selected from those treated at BC Cancer between January 2017 and 31 May 2019. Exclusions applied to those who received first-line, other immunotherapies, radiation without assessable response, had autoimmune diseases, or were on long-term immunosuppressants. All 114 patients underwent a baseline CT scan 6 weeks prior to treatment and a follow-up CT at 9–12 weeks after starting Pembrolizumab therapy. The cohort was divided into a discovery set (*n* = 97, 56F/41M, age 73 ± 6, consisting of those patients referred before December 2018) and a test set (*n* = 17, 9F/8M, age 65 ± 10, consisting of patients referred after January 2019), as illustrated in Figure 1; a comparison of clinical descriptors can be found in Table 1.

Baseline and follow-up CT imaging was performed using a GE Light Speed CT scanner (GE Healthcare, Milwaukee, WI, USA) under standard institutional imaging protocols, as previously described [23]. Images were reconstructed with a ‘soft’ kernel (i.e., ‘Standard’ or ‘B35f’ equivalent), slice thickness of 2–3 mm, and an average in-plane resolution of 0.72 × 0.72 mm. Standard lung window settings (level −600 HU, width 1500 HU) were applied, followed by intensity rescaling to an 8-bit range (0–255) for subsequent analysis. Two board-certified radiologists independently reviewed all scans while blinded to outcomes; discrepancies were resolved by consensus. Baseline assessments included measuring the dominant lung lesion and evaluating the number of metastatic sites as an estimate of overall tumor burden. Supplementary imaging studies, such as PET, MRI, or ultrasound with biopsy information when available, were used to confirm metastatic disease.

Treatment response was evaluated using the response evaluation criteria in solid tumors (RECIST v1.1) [25]. Progressive Disease (PD) corresponded to a ≥20% increase in total lesion diameter. Complete Response (CR) referred to the disappearance of all target lesions. Partial Response (PR) indicated a ≥30% reduction in lesion diameter, whereas Stable Disease (SD) included cases that did not satisfy the criteria for either PR or PD. Patients grouped as PD were split into two sets for the training set (27 patients, 31 lesions, 714 slices) and for the test set (9 patients, 9 lesions, 324 slices); the disease control (DC) set included CR, PR, or SD, and was split in two sets as well: training set (70 patients, 88 lesions, 1466 slices) and test set (8 patients, 10 lesions, 163 slices). Figure 1 summarizes the distribution of patient-, lesion-, and slice-level samples included in the discovery training and preliminary test cohorts. Overall, 21 patients from both cohorts contributed more than one target lung lesion to the study.

### 2.2. CT Baseline Clinical Descriptors and Risk Score Predictor

Clinical and demographics information was collected at the baseline CT time point for both discovery and preliminary test cohorts, including age, sex, disease stage (stage III or stage IV), Eastern Cooperative Oncology Group (ECOG) score, target lesion size (lesion diameter), smoking status, pack-years smoked, and the number of sites with metastases (tumor burden/number of involved organs) [26]. Table 1 includes the 16 clinical descriptors that were considered in this study. A separate retrospective study shows that tumor burden and smoking pack-year history are distinct indicators of PD to pembrolizumab monotherapy [23]. To enhance model generalizability, we performed standardized preprocessing for all features. Categorical variables (e.g., sex, smoking status) were converted into binary indicators using one-hot encoding, while other variables were standardized (divided by their mean value) and adjusted based on scaling parameters learned from the discovery dataset. These same scaling parameters were then applied directly to the preliminary test set to prevent information leakage. A statistical test (Mann–Whitney U) was applied to the 16 clinical features to rank them. Finally, we employed a linear support vector machine (SVM) model as a risk score predictor to calculate a clinical progressive risk score, which we made use of for later analysis [16,17,21,22]. By analyzing the area under the ROC curve (AUC-ROC) and F1 metrics for models using all 16 descriptors and a reduced number of descriptors, we determined whether a reduced feature set was sufficient for integration with the deep learning framework.

### 2.3. Lung Lesion Annotation and ROI Segmentation

All discrete lung lesions identified on the baseline CT scans were annotated by the same reviewing radiologist using soft-tissue windows settings. A volume of interest (VOI) was annotated (by LD or IJ) and confirmed by a radiologist (RY), delineating bounding boxes on axial images of the diagnosed lesion region of interest (ROI) as seen on the patient’s CT scan. We captured the lesions as a set of square 2D ROI of 128 by 128 pixels for all the slices in the VOIs. For each lesion, we utilized a minimum of five axial slices to provide pseudo-volumetric information for deep learning-based feature extraction. This included the largest tumor slice identified by the radiologists, together with at least four adjacent axial view slices. We captured a total of 119 lesions (mean: 17 slices/lesion) from 97 patients (mean: 21 slices/patient) for the discovery training set and a total of 19 lesions (mean: 29 slices/lesion) from 17 patients (mean: 21 slices/patient) for the preliminary test set (Figure 1).

### 2.4. Deep Learning Feature Extraction

**Figure 2:**
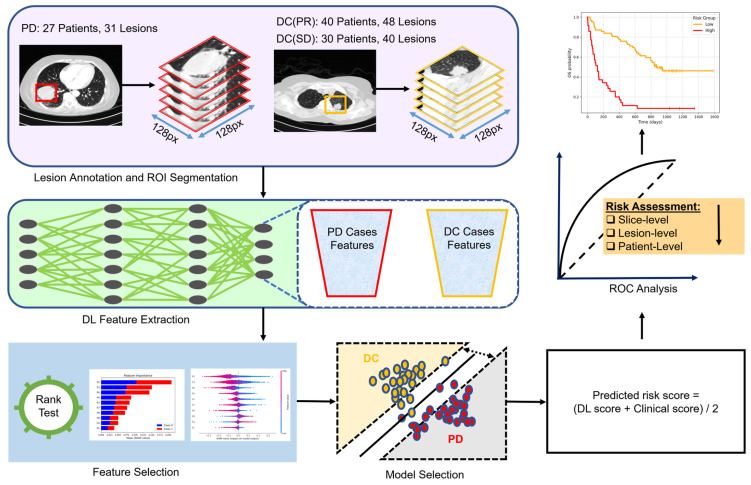
Overview of the proposed AI framework for predicting response to pembrolizumab monotherapy in advanced non–small cell lung cancer. Square ROIs (128 × 128 pixels) were segmented from lesions in progressive disease (PD) and disease control (DC) patients and used to train a deep learning model for feature extraction. A statistical rank-based test (Mann–Whitney U) was applied to select top-ranked features, and their importance was examined using an explainable AI tool (SHAP). Risk scores were computed by averaging deep learning-derived and clinical scores and were analyzed using ROC analysis at three levels (slice, lesion, and patient). Patient survival stratification (high- vs. low-risk) was performed based on model-predicted outcomes. Partial response (PR) and stable disease (SD) indicate patients exhibiting partial response and stable disease, respectively.

The discovery training set included 27 patients in the PD group and 70 patients in the DC group, consisting of 40 PR patients and 30 SD patients (Figure 1). To preserve data balance and enable transfer learning, this dataset was divided into two subgroups (group A and group B), which were used in a two-phase training strategy using the VGG16 model [27]. The VGG16 model was trained on group A: PR (40 patients, 564 ROIs) vs. PD (27 patients, 563 ROIs). In all the training phases (Figure 2), input ROIs were augmented to reduce the overfitting using the automatic Keras image data generator (TensorFlow v2.16.1, Google Brain Team, Mountain View, CA, USA), which included rotation, zoom, flipping, and shifting. A feedforward multi-layer perceptron (MLP) was used as the classifier on top of the VGG16 feature extractor. The MLP was composed of two hidden layers followed by a prediction layer with softmax activation. Model training employed the Adam optimizer with a learning rate set to 1 × 10^−5^ and a batch size of 32. Categorical cross-entropy was applied to monitor training/validation accuracy and loss across epochs. To avoid selection bias, a randomized cross-fold of patients from the training data, denoted as the validation set (*n* = 20% patients), was set aside prior to any model or feature selection. To mitigate inadvertent information leakage, the discovery training and preliminary test sets were partitioned. Model selection and feature extraction were performed only on the training/validation data, while the preliminary test set was completely held out and not used in any part of the model development or hyperparameter tuning.

In the second training phase, transfer learning was applied by reusing the feature representations learned during the first phase. Specifically, the convolutional layers of the trained VGG16 model were frozen, while the original MLP classifier was replaced with a newly initialized MLP. The new MLP, with weights set to zero, was retrained on group B to differentiate the SD group (30 patients, 575 ROIs) from the PD group (27 patients, 563 ROI) (Figure 2). During this phase, only the MLP layers were updated, allowing the model to adapt the previously learned image representations to the new classification task. The best-performing model from the second training phase was selected based on validation performance and subsequently used to extract features from both discovery training and testing sets (Figure 1). To obtain a compact and linearly discriminative representation, the penultimate layer of the trained VGG16–MLP model was removed, and a 128-dimensional feature vector that made up its inputs was extracted from each ROI slice (Figure 2). All model construction, training, and feature extraction were implemented using the Keras deep learning framework (TensorFlow v2.16.1).

### 2.5. Feature Selection and Risk Score Predictor

**Figure A1:**
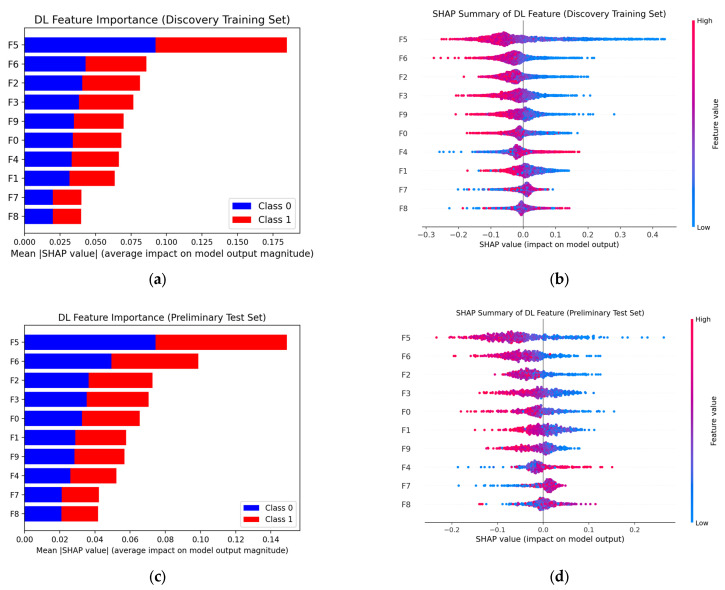
SHAP analysis of 10 deep learning features’ impact (bar plots) and influence (summary plots) on model predictions: (**a**,**b**) discovery training set and (**c**,**d**) preliminary test set. Across both datasets, feature F5 consistently exhibits the strongest contribution, whereas F8 shows the weakest. The summary plots offer a comprehensive visualization of how the seven deep learning features collectively influence the support vector machine decision-making process.

We employed the Mann–Whitney U test (SciPy v1.17.0), a non-parametric test, to rank the individual 128 DL features’ ability to differentiate between the PD and DC patients in the training set. Then we used the (7–10) highest-ranked features to train two types of SVM classifiers, (i) SVM with radial basis function kernel (SVM-RBF) and (ii) SVM with Polynomial kernel (SVM-Poly). Additionally, the SHapley Additive exPlanations (SHAP) tool (v0.50.0) demonstrated the impact and influences of DL features in Appendix A, Figure A1.

To avoid model selection bias, the performance of SVM-RBF and SVM-Poly models was evaluated on the discovery set using ROC analysis and F1 scores (Figure 2) at the slice, lesion, and patient levels. The patient-level analysis was considered the primary clinically relevant endpoint, while slice- and lesion-level analyses were included as intermediate representations within the proposed multi-level framework. We selected the model that demonstrated the most consistent and superior performance across all three levels. This final model was subsequently applied to the preliminary test set to predict individual risk scores per slice.

At the slice level, multiple axial slices were evaluated per lesion; a slice was classified as progressive only if its risk score exceeded cutoff values calculated using Youden’s J statistic from the ROC analysis on the training data.

To derive lesion-level scores from slice-level predictions, only the top 30% highest-scoring slices per lesion were considered [28,29]. A lesion was then classified as progressive if at least two adjacent slices exceeded the average score of this top 30% subset. This was chosen as a slight denoising step, as the likelihood that two adjacent slices will exceed the average score and be a false-positive should be lower (as they are potentially measuring the same aggressive tumor area) than any two slices above the threshold not matching this adjacency criteria. Both training and test datasets included patients with one or more lesions; for example, if a patient has three lesions, then the final progression score for this patient was defined as the maximum score among all the lesions.

The performance of each classifier was compared with and without the inclusion of the derived clinical risk score, as outlined in Section 2.2. The selected clinical features were used to compute the clinical risk score. The deep learning model generated a probabilistic output from the SVM classifier. When incorporating clinical descriptors, the final risk score at each level was computed using equal-weight late fusion by averaging the probabilistic output of the deep learning SVM and the clinical risk score.

Progression risk prediction for preliminary test patients was evaluated using ROC and F1 scores. Confidence intervals (CIs) for AUC values were estimated using a non-parametric bootstrap method [30,31]. Train/test partitioning was performed strictly at the patient level to avoid overlap of slices or lesions between cohorts. Overall model performance on the discovery training and preliminary test sets was compared using AUC, sensitivity (SN), specificity (SP), and F1 score. “Low-risk” and “high-risk of progression” groups were then defined based on predicted progression disease risk scores (PDRS), derived from combined clinical and deep learning (Clinical + DL) features using a three-level SVM-based analysis (slice, lesion, patient). Progression-free survival (PFS) and overall survival (OS) were compared between high- and low-risk groups using Kaplan–Meier survival analysis. Patients were stratified based on a risk score threshold (Youden index cutoff = 0.4) derived from the training cohort, with those above the cutoff assigned to the high-risk group and those below the cutoff assigned to the low-risk group [32]. In this study, OS was defined as the time from initiation of pembrolizumab monotherapy to death from any cause or last clinical follow-up. The PFS was defined as the time from initiation of pembrolizumab monotherapy to radiologically confirmed disease progression according to RECIST v1.1 criteria, death, or the last clinical follow-up.

## 3. Results

### 3.1. Clinical Risk Score Analysis

**Figure 3:**
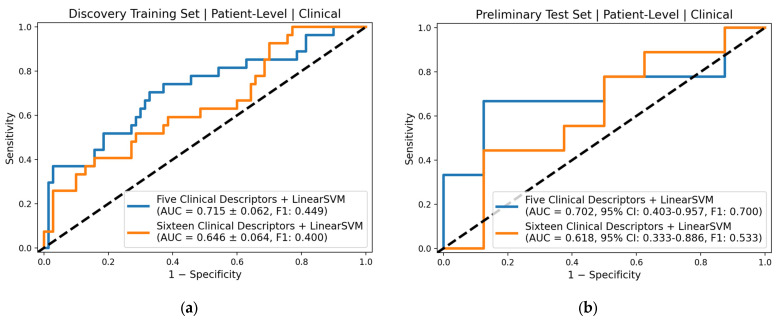
ROC analysis at the patient-level was performed to evaluate the performance of the Linear SVM using all 16 clinical features (orange) versus the five selected clinical features (blue) on both the discovery set (**a**) and preliminary test set (**b**), with training conducted on the discovery set. The model demonstrated improved AUC (mean ± SD) and F1 scores when trained on the five selected features.

**Figure A2:**
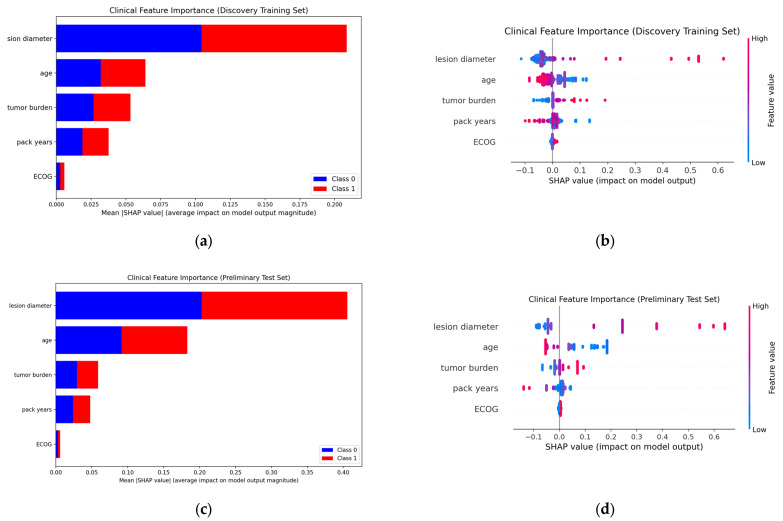
SHAP analysis of five clinical feature impact (bar plots) and influence (summary plots) on model predictions: (**a**,**b**) discovery training set and (**c**,**d**) preliminary test set. Across both datasets, the five clinical features exhibit a consistent ordering of importance, with the ECOG score contributing the least. The summary plots illustrate how these clinical features collectively influence the predictions of the linear support vector machine.

A Linear SVM was used to estimate risk scores and compare performance between selected clinical features and the full set of 16 clinical features. We found the five most significant (*p*-values < 0.05) descriptors, which included ‘lesion diameter’, ‘pack years’, ‘age’, ‘ECOG’, and ‘tumor burden’ (Appendix A Table A1). The model showed good performance with five features: AUC of 0.72 ± 0.06 and F1 score of 0.45 on the discovery set; AUC of 0.70 (95% CI: 0.403–0.957) and F1 score of 0.70 on the preliminary test set, as shown in Figure 3a,b. As a result, we only used five descriptors to calculate the clinical risk of progression. The SHAP values illustrated the impact and influences of five clinical features in Appendix A, Figure A2.

### 3.2. Discovery Training Set: Slice-Level Classification Performance

**Figure 4:**
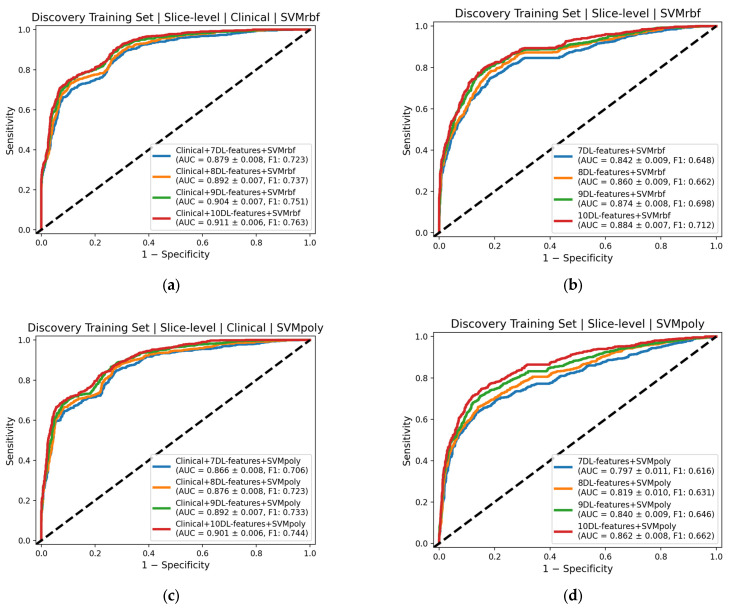
Comparison of slice-level classification performance, (**a**,**c**) with and (**b**,**d**) without clinical features using the SVM-RBF and SVM-Poly classifiers. Inclusion of clinical features consistently improves both AUC and F1 scores across models with 7–10 deep learning (DL) features.

At the slice level, incorporating clinical features alongside deep learning (DL) features consistently improved model performance using the SVM-RBF classifier. The clinical score acts as a patient-specific offset for all the slices from that patient, providing the same boost to all the slices from that patient (another way would be to allow a patient-specific adjusted threshold for each patient based upon their clinical score). As the number of DL features increased from 7 to 10, both AUC and F1 scores improved across all configurations. The best performance was observed with the combination of clinical features and 10 DL features, yielding an AUC of 0.911 ± 0.006 and an F1 score of 0.763 on the training data (Figure 4a). In contrast, models trained without clinical features showed comparatively lower performance, with the highest results achieved using 10 DL features alone (AUC: 0.884 ± 0.007, F1: 0.712) (Figure 4b). These findings highlight the added predictive value of clinical information in enhancing slice-level classification accuracy.

A similar trend was observed with the SVM-Poly classifier: integrating clinical features alongside deep learning (DL) features consistently enhanced slice-level classification performance. As the number of DL features increased from 7 to 10, performance steadily improved. The combination of clinical data with 10 DL features achieved the highest metrics (AUC: 0.901 ± 0.006, F1: 0.744) (Figure 4c), closely aligning with the performance seen in the SVM-RBF model.

In contrast, models trained without clinical features underperformed across all DL feature configurations. The best result without clinical information (10 DL features) reached an AUC of 0.862 ± 0.008 and an F1 of 0.662, which was notably lower than the corresponding configuration with clinical input (Figure 4d). These findings further confirm the value of incorporating clinical descriptors to boost predictive accuracy, regardless of kernel choice.

### 3.3. Discovery Training Set: Lesion-Level Classification Performance

**Figure 5:**
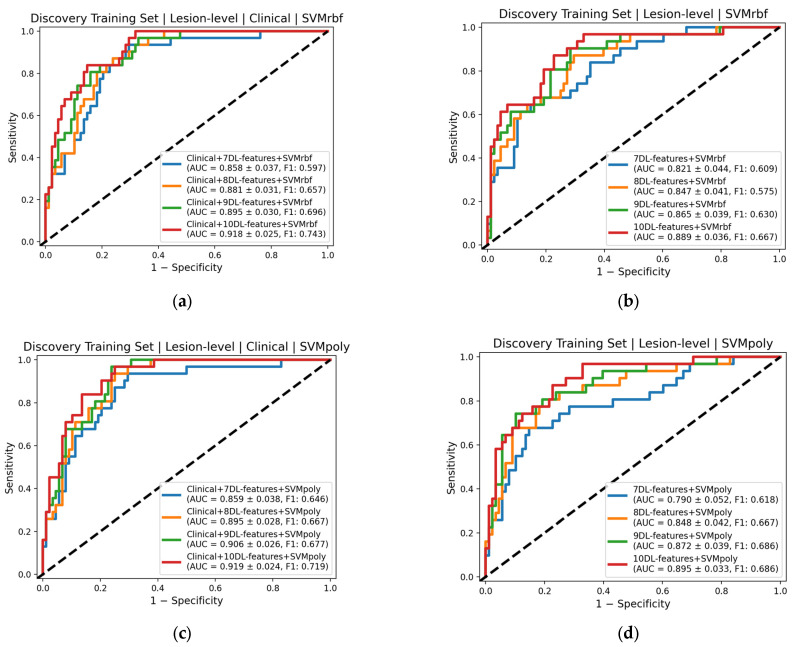
Comparison of lesion-level classification performance, (**a**,**c**) with and (**b**,**d**) without clinical features using the SVM-RBF and SVM-Poly classifiers. Inclusion of clinical features consistently improves both AUC and F1 scores across models with 7–10 deep learning (DL) features.

At the lesion level, the inclusion of clinical features continued to enhance model performance when combined with deep learning (DL) features. As seen at the lesion level, both AUC and F1 scores improved steadily with the increasing number of DL features. The best performance with SVM-RBF was achieved by Clinical + 10 DL features, resulting in an AUC of 0.918 ± 0.025 and an F1 score of 0.743 (Figure 5a), demonstrating strong lesion-level discrimination.

In contrast, the same model without clinical features showed lower performance. Even the best non-clinical configuration (10 DL features) reached only AUC: 0.889 ± 0.036 and F1: 0.667 (Figure 5b), underscoring the added value of integrating clinical descriptors. Interestingly, while the non-clinical model with 7 DL features had a relatively higher F1 (0.609) compared to the clinical counterpart (0.597), it still underperformed in AUC.

Consistent with findings from the SVM-RBF analysis, the SVM-Poly classifier also demonstrated improved lesion-level performance when clinical features were included alongside deep learning (DL) features. The model’s predictive power increased with the number of DL features, achieving the best results with Clinical + 10 DL features, yielding an AUC of 0.919 ± 0.024 and an F1 score of 0.719 (Figure 5c).

In comparison, models trained without clinical features showed inferior performance across all configurations. Even the strongest non-clinical setup (10 DL features) achieved a slightly lower AUC of 0.895 ± 0.033 and F1 score of 0.686 (Figure 5d), reaffirming the incremental value of clinical descriptors. Notably, the non-clinical model with 9 DL features reached an identical F1 score (0.686) as the 10-DL setup but still trailed in AUC, suggesting diminishing returns without clinical input.

### 3.4. Discovery Training Set: Patient-Level Classification Performance

**Figure 6:**
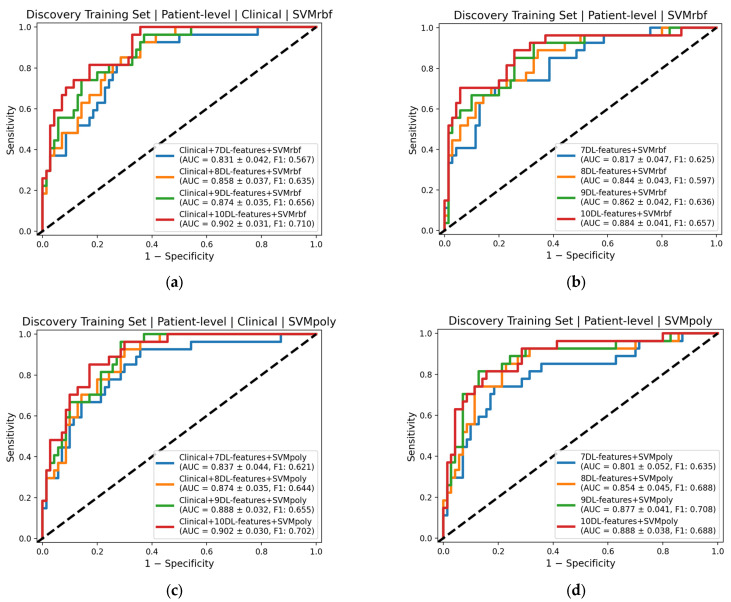
Comparison of patient-level classification performance, (**a**,**c**) with and (**b**,**d**) without clinical features using the SVM-RBF and SVM-Poly classifiers. Inclusion of clinical features consistently improves both AUC and F1 scores across models with 7–10 deep learning (DL) features.

For the patient-level outcome—our objective for clinical relevance and diagnostic decision-making—the SVM-RBF classifier continued to show enhanced predictive performance when clinical features were integrated. As with prior levels, increasing the number of deep learning (DL) features led to steady improvements in both AUC and F1 scores. The best results were achieved using Clinical + 10 DL features (Table 2), with an AUC of 0.902 ± 0.031 and an F1 score of 0.710 (Figure 6a), indicating robust patient-level classification.

In contrast, models trained without clinical features yielded consistently lower performance. Even the strongest configuration without clinical data (10 DL features) reached only AUC = 0.884 ± 0.041 and F1 = 0.657 (Figure 6b), underscoring the added predictive value of clinical descriptors in patient-level assessment. Notably, while some configurations without clinical data showed reasonable performance (e.g., 7 DL features had F1 = 0.625), they lagged behind in overall discriminatory power.

At the patient level, the SVM-Poly classifier demonstrated progressively improved performance with the addition of more deep learning (DL) features. The highest predictive accuracy was achieved using Clinical + 10 DL features, resulting in an AUC of 0.902 ± 0.030 and an F1 score of 0.702 (Figure 6c). These results closely align with those of the SVM-RBF classifier, confirming the robustness of the 10-feature model across kernel choices. Interestingly, models without clinical features showed an AUC of 0.877 ± 0.041 and 0.888 ± 0.038; F1 scores of 0.708 and 0.688, with 9 and 10 DL features (Figure 6d). However, even these results are better than clinical plus 7DL and/or 8DL features.

Among the evaluated classifiers, SVM-RBF with 10 DL features plus clinical, consistently demonstrated superior and more stable performance across all analysis levels—slice, lesion, and patient (Table 2). Although the difference in AUC between SVM-RBF and SVM-Poly at the patient level was modest, SVM-RBF achieved higher F1 scores and exhibited reduced performance variability, particularly at the patient level, where clinical decision-making is most critical. This indicates a better trade-off between sensitivity and specificity, which is vital for the reliable prediction of progressive disease. Owing to its consistent advantage across key metrics and its robustness in modeling, SVM-RBF was selected as the final model for the subsequent test cohort in PD outcome prediction.

### 3.5. Preliminary Test Set: Patient-Level Classification Performance Using SVM-RBF Classifier

**Figure 7:**
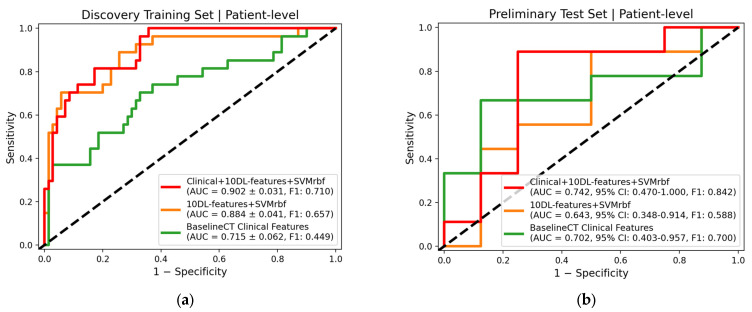
Patient-level performance of SVM-RBF classifier using clinical, deep learning, and combination features: (**a**) discovery training set and (**b**) preliminary test set. AUC values are reported as mean ± standard deviation for the train set and with 95% confidence intervals for the test set. F1 scores indicate classification accuracy for progressive disease prediction.

To conclude the model selection process, we performed a final comparative analysis using the top-performing classifier, SVM-RBF, across three input configurations—clinical features only, DL features only, and their combination—evaluated at the patient level (Table 2). On the training set, the integrated model using clinical features and the top 10 DL features yielded the highest performance (AUC = 0.902 ± 0.031; F1 = 0.710), outperforming both the DL-only model (AUC = 0.884 ± 0.041; F1 = 0.657) and the clinical-only model (AUC = 0.715 ± 0.062; F1 = 0.449) (Figure 7a).

This superiority was also demonstrated in the preliminary test set (Table 2), where the combined model again achieved the highest F1 score (0.842) and a favorable AUC (0.742; 95% CI: 0.470–1.000), outperforming both the DL-only (AUC = 0.643; F1 = 0.588) and clinical-only (AUC = 0.702; F1 = 0.700) models (Figure 7b). These findings reinforce the complementary value of combining imaging-derived DL features with baseline clinical descriptors and further confirm SVM-RBF as the most effective model for reliable patient-level progressive disease prediction in preliminary validation.

After computing the progressive disease risk score (PDRS) for each axial slice of a lung lesion, slices were classified as either “low risk” or “high risk of progression” based on whether their score fell below or above the predefined cutoff of 0.4. To determine progression, we applied a thresholding approach guided by the discovery set. A lesion was classified as PD if at least two of its adjacent axial slices had risk scores above the predefined cutoff. At the patient level, having just one such lesion was sufficient to label the patient as progressive.

The SVM-RBF’s performance was evaluated by stratifying predictions into high-risk and low-risk groups at the slice, lesion, and patient levels (Table 3). On the training set, high-risk groups demonstrated strong sensitivity across all levels, with 71.4% of progressive slices (510/714), 93.9% of progressive lesions (26/31), and 81.5% of progressive patients (22/27) correctly identified. Correspondingly, low-risk groups exhibited strong specificity, correctly identifying 92.4% of controlled slices (1354/1466), 85.2% of controlled lesions (75/88), and 81.4% of controlled patients (57/70). On the preliminary test set, the model maintained solid generalizability. It correctly identified 88.9% of progressive lesions and patients (8/9), while the low-risk group accurately captured 70.0% of controlled lesions (7/10) and 75.0% of controlled patients (6/8). Although slice-level sensitivity dropped in the test set (45.7%, 148/324), specificity remained high (82.2%, 134/163).

**Figure 8:**
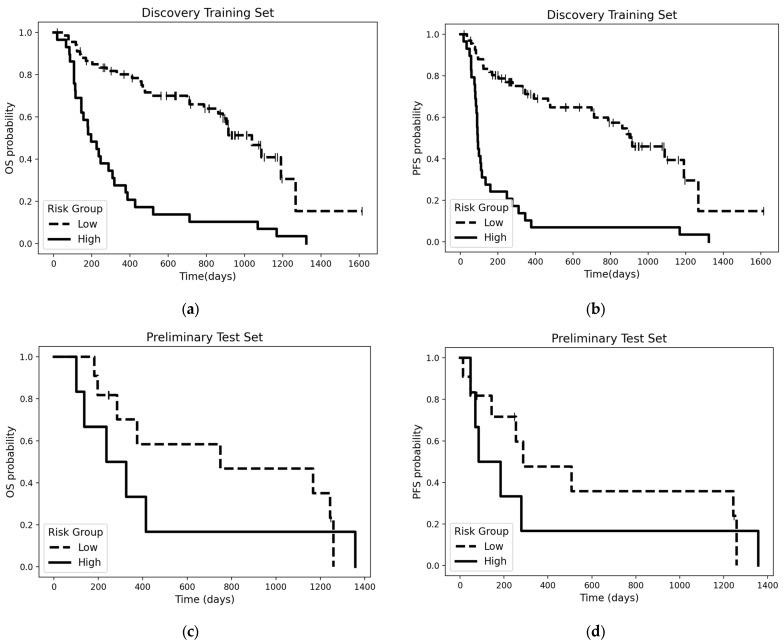
Kaplan–Meier curves for overall survival (OS) and progression-free survival (PFS) in the (**a**,**b**) discovery training set and (**c**,**d**) preliminary test set, using a risk-stratified threshold derived from the model-generated risk score (RS). Patients were classified as high- or low-risk using the Youden cutoff (RS > 0.4) determined from the discovery training set.

Risk stratification using the model-derived risk score showed clear separation in both discovery and preliminary test cohorts for OS and PFS, based on a risk-stratified threshold analysis (Figure 8a–d). In the training cohort, high-risk patients consistently surpassed the threshold, whereas a subset of low-risk patients (*n* = 37) remained below the threshold (Figure 8a,b). The preliminary test cohort showed a similar pattern, with high-risk patients exceeding the threshold, while only a small subset of low-risk patients (*n* = 3) remained below it (Figure 8c,d). Overall, these findings suggest that the risk score provides consistent prognostic discrimination across cohorts and survival endpoints, although late survival estimates should be interpreted with caution, given the limited number of patients remaining at risk at extended follow-up time points in the preliminary test cohort.

## 4. Discussion

In this study, we developed an artificial intelligence (AI) model to predict progressive disease (PD) following first-line pembrolizumab monotherapy, a standard treatment scenario in oncology practice, in patients with advanced non-small cell lung cancer (NSCLC) characterized by high PD-L1 expression and the absence of EGFR or ALK mutations. The proposed model leverages a hybrid AI framework that integrates deep learning-derived CT imaging features with key clinical variables, including tumor diameter, smoking history (pack-years), age, ECOG performance status, and tumor burden. Our findings underscore the potential of deep learning-based imaging biomarkers to support early risk stratification and guide more personalized first-line treatment strategies in this clinically relevant patient population.

While previous DL-based immunotherapy studies have shown that DL features complement clinical, genomic, and metastasis variables, and that selective DL feature subsets can improve model stability, these approaches have several limitations [14,15,16,17,18,19,20,21,22]. The study on PET/CT features used single-level evaluation schemes or lacked rigorous external testing [19,20]. Unlike these prior DL efforts, our DL study introduces a multi-level evaluation framework, assessing model performance at the slice, lesion, and patient levels to capture what we assume to be biologically meaningful tumor heterogeneity, though we do not have a second biological resolution (i.e., pathology slides) available to us to confirm this speculation. The slight drop in AUC from lesion-level to patient-level (0.919 vs. 0.902 for SVM-RBF using 10 DL + clinical features) likely reflects the personalized oncological nature of the method: while lesion-level predictions likely capture local tumor or peritumoral heterogeneity with high fidelity, patient-level aggregation introduces variability from multiple lesions and slices, slightly reducing overall discriminative performance. Importantly, this approach enables both detailed lesion-level interpretation—potentially guiding biopsy or targeted monitoring—and overall patient-level risk assessment, supporting more personalized treatment strategies. Additionally, by implementing feature-selection and model-selection procedures, we identify a more stable classifier across all levels rather than relying on performance at a single resolution. While our study builds upon the same patient cohort used in [24], the present study integrates DL-derived imaging features with clinical data. Finally, validation on the preliminary test cohort demonstrates the generalizability of our approach, achieving a balanced trade-off between sensitivity and specificity, along with an improving F1 score as we ascend from slice- to patient-level predictions. Collectively, these innovations underscore the potential of this method as an adjunct tool to support timely, personalized disease management in advanced NSCLC.

Our study design differs from a conventional single train/test split in that the discovery cohort was partitioned into subgroups (PR vs. PD for phase 1; SD vs. PD for phase 2) to enable a structured transfer-learning framework. This grouping is aligned with RECIST v1.1 criteria, where PR and PD represent distinct response extremes, while SD reflects an intermediate and clinically heterogeneous category that does not meet criteria for either response or progression. By first training the model to distinguish clear responders (PR) from progressors (PD), and subsequently refining classification for SD versus PD, the model is guided to learn clinically meaningful progression patterns. This multi-phase strategy mirrors real-world oncological decision-making, where distinguishing non-responders is critical and intermediate cases require more nuanced assessment. During each training phase, a randomized hold-out validation set was used to monitor model performance and prevent overfitting. More importantly, model generalizability was evaluated using an independent preliminary test cohort, which provides a stronger assessment of robustness than internal cross-validation alone, reducing optimistic bias and better reflecting real-world performance.

This study’s strength lies in the consistent clinical characteristics of the patient cohort (Table 1). However, several limitations exist. Most patients were Caucasian and treated at a single center, which may limit the model’s generalizability to more diverse populations—a common issue in North American lung cancer datasets [33]. The small sample size also restricted our ability to evaluate the impact of racial variation. Additionally, we used 1D deep learning-based features, which differ from traditional radiomic features. We reduced the dimensionality of the imaging features from 128 to 10 features via feature selection. The clinical feature selection was also based on individual statistical testing in the discovery cohort, which may introduce instability due to the limited number of events and absence of correction for multiple hypothesis testing. Rather than using the full feature set, combining this selected subset together with clinical variables improved classifier performance and promoted model diversity, consistent with observations reported in prior studies [18,20,22]. Despite this, our multi-level approach (from slice to patient) supports the potential of this method as an additional tool, in parallel to the radiomic signature, to help identify non-responders to anti-PD-1 therapy in an ensemble prediction framework. While cross-validation is commonly recommended for small datasets to reduce sampling bias, we stayed with the more conservative training, validation, and test design as it is less likely to overestimate the method’s performance on a new dataset while keeping the same patient data split we previously described for comparability with [24]. This may also contribute to variability in feature selection and performance estimates across cohorts. A limitation of this study is that a simple equal-weight averaging strategy was used for multimodal fusion. More advanced ensemble methods, such as weighted fusion or stacking-based approaches, may further improve predictive performance in future work.

To address additional limitations of this study, future work will propose three key directions. First, advanced generative adversarial networks (GANs) will be used to generate synthetic training data, helping to improve model performance while avoiding data leakage [34,35,36,37,38,39,40]. Second, the relatively wide confidence intervals (0.470–1.000) observed in the preliminary test cohort likely reflect the limited sample size and class distribution, which may introduce instability and reduce the precision of the reported performance estimates. In addition, future studies should incorporate more robust feature-selection strategies (e.g., resampling-based or regularized approaches) to improve the stability of clinically selected variables. Therefore, larger prospective multi-center studies are warranted to better assess the robustness and generalizability of the proposed approach. Third, integrating CT-derived radiomic and DL features with detailed pathological information, such as tumor cell morphology and characteristics of the tumor microenvironment, may further enhance the accuracy and clinical relevance of the prediction model.

Overall, these results support the model’s effectiveness in distinguishing patients at risk of progression, particularly at the lesion and patient levels, which are most relevant for clinical decision-making.

## 5. Conclusions

This study introduces an AI-based prediction tool that integrates deep learning-derived CT features with clinical data to identify advanced NSCLC patients with high PD-L1 expression who are unlikely to benefit from first-line pembrolizumab monotherapy. By applying a multi-level progressive disease risk score analysis across slices, lesions, and patients, the model demonstrates improved patient-level predictions of treatment response. These findings provide preliminary evidence supporting future prospective studies of AI-assisted treatment stratification in advanced NSCLC patients receiving pembrolizumab-based therapy.

## Figures and Tables

**Table 1:** Baseline CT clinical descriptors of patients in the discovery training set and the preliminary test set.

Clinical Descriptors	Discovery Training Set (*n* = 97)		Preliminary Test Set *(n* = 17)	
	Progressive Disease (PD) *n* = 27	Disease Control (DC) *n* = 70	Progressive Disease (PD) *n* = 9	Disease Control (DC) *n* = 8
Age, mean (min–max)	70 (65–76)	74 (70–79)	63 (57–67)	64 (59–74)
Sex (*n*, %):				
Female	14 (51.9)	42 (60)	6 (66.7)	3 (37.5)
Male	13 (48.1)	28 (40)	3 (33.3)	5 (62.5)
Smoking Status (*n*, %):				
Light and non-smoker	2 (7.4)	2 (2.9)	0 (0)	1 (12.5)
Smoker (current/ex)	24 (88.9)	65 (92.8)	9 (100)	7 (87.5)
Unknown	1 (3.7)	3 (4.3)	0 (0)	0 (0)
Pack years, mean (min–max)	33 (20–49)	45 (27–50)	32 (25–40)	51 (32–68)
Cancer subtype (*n*, %):				
Non-SCC	22 (81.5)	58 (82.8)	4 (44.4)	6 (75)
SCC	5 (18.5)	8 (11.4)	5 (55.6)	2 (25)
Not specified	0 (0)	4 (5.7)	0 (0)	0 (0)
ECOG Score, mean (%):				
≤1	13 (48.1)	39 (55.7)	4 (44.4)	7 (87.5)
>1	12 (44.4)	22 (31.4)	5 (55.5)	1 (12.5)
Unknown	2 (7.4)	9 (12.8)	0 (0)	0 (0)
Disease stage (*n*, %):				
III	1 (3.7)	13 (18.6)	0 (0)	0 (0)
IV	26 (96.3)	57 (81.4)	9 (100)	8 (100)
Metastatic (*n*, %):				
Lung Metastasis	16 (59.3)	40 (57.1)	5 (55.5)	6 (75)
Lymph Node	24 (88.9)	55 (78.6)	9 (100)	3 (37.5)
Adrenal	10 (37)	10 (14.3)	4 (44.4)	0 (0)
Liver	5 (18.5)	5 (7.1)	1 (11.1)	0 (0)
Bone	14 (51.8)	18 (25.7)	3 (33.3)	1 (12.5)
Brain	6 (22.2)	21 (30)	2 (22.2)	3 (37.5)
Other	14 (51.8)	30 (42.8)	3 (33.3)	0 (0)
Tumor Burden, mean (min–max)	3 (2–4)	3 (2–3)	3 (3–5)	2 (1–3)
Lesion diameter (mm), mean (min–max)	39.9 (24.5–51.5)	36 (24.2–49.2)	47 (28–50)	55 (25.5–84.5)

**Table 2:** Patient-level AUC, sensitivity (SN), specificity (SP), and F1 comparison of the SVM-RBF classifier across discovery training (*n* = 97) and preliminary test (*n* = 17) sets using three feature configurations: clinical features only, deep learning (DL) features only, and combined clinical + DL features.

	Discovery Train Set (*n* = 97 Patients)				Preliminary Test Set (*n* = 17 Patients)			
Feature Type	AUC	SN	SP	F1	AUC	SN	SP	F1
Clinical Features	0.715 ± 0.062	0.407	0.843	0.449	0.702 (CI 95%: 0.403–0.957)	0.778	0.500	0.700
DL Features	0.884 ± 0.041	0.815	0.743	0.657	0.643 (CI 95%: 0.348–0.914)	0.556	0.625	0.588
Clinical + DL Features	0.902 ± 0.031	0.815	0.814	0.710	0.742 (CI 95%: 0.470–1.000)	0.889	0.750	0.842

**Table 3:** Comparison of discovery and preliminary test set performance for patient-, lesion-, and slice-level progressive disease risk prediction using the SVM-RBF classifier.

		**Discovery Train Set**		**Preliminary Test Set**	
		Patient-Level			
	True Response (PD vs. DC)	PD (*n* = 27)	DC (*n* = 70)	PD (*n* = 9)	DC (*n* = 8)
Risk of progression					
High-risk Patient		22	13	8	2
Low-risk Patient		5	57	1	6
		SN: 0.815	SP: 0.814	SN: 0.889	SP: 0.750
		Lesion-Level			
	True Response (PD vs. DC)	PD (*n* = 31)	DC (*n* = 88)	PD (*n* = 9)	DC (*n* = 10)
Risk of progression					
High-risk Lesion		26	13	8	3
Low-risk Lesion		5	75	1	7
		SN: 0.939	SP: 0.852	SN: 0.889	SP: 0.700
		Slice-Level			
	True Response (PD vs. DC)	PD (*n* = 714)	DC (*n* = 1466)	PD (*n* = 324)	DC (*n* = 163)
Risk of progression					
High-risk Slice		510	112	148	29
Low-risk Slice		204	1354	176	134
		SN: 0.714	SP: 0.924	SN: 0.457	SP: 0.822

## Data Availability

The data generated or analyzed during the study are available from the corresponding author upon request.

## Abbreviations

The following abbreviations are used in this manuscript:

ALK	Anaplastic lymphoma kinase
AI	Artificial intelligence
AUC	Area under the curve
BRAF	A human gene that encodes a protein called B-Raf
CIs	Confidence intervals
CR	Complete response
CT	Computerized tomography
DC	Disease control
DL	Deep learning
ECOG	Eastern Cooperative Oncology Group
EGFR	Epidermal growth factor receptor
ICIs	Immune checkpoint inhibitors
KRAS	Kirsten rat sarcoma virus
Linear SVM	SVM with linear kernel
MRI	Magnetic resonance imaging
NSCLC	Non-small cell lung cancer
NTRK	Neurotrophic tyrosine receptor kinase
HU	Hounsfield unit
OS	Overall survival
PD	Progressive disease
PDRS	Progressive disease risk score
PET	Positron emission tomography
PFS	Progression-free survival
PR	Partial response
RECIST	Response evaluation criteria in solid tumors
ROC	Receiver operating characteristic
SCC	Squamous cell carcinoma
SD	Stable disease
SN	Sensitivity
SP	Specificity
SVM	Support vector machine
SVM-RBF	SVM with radial basis function kernel
SVM-Poly	SVM with polynomial kernel

## Appendix A

**Table A1:** A list of 16 clinical descriptors with *p*-values from the discovery train set.

16 Clinical Features Name	*p*-Value (Discovery Train Set)
Age	0.0236
Sex	0.2935
Smoking status	0.6930
Pack years	0.0135
Cancer subtype	0.1483
ECOG score	0.0362
Disease stage	0.0632
Lung metastasis	0.8508
Lymph node	0.2438
Adrenal	0.0641
Liver	0.1004
Bone	0.1697
Brain	0.5661
Other	0.5107
Tumor burden	0.0466
Lesion diameter	0.0094
